# Mitochondrial genome of the Silvertip shark, *Carcharhinus albimarginatus,* from the British Indian Ocean Territory

**DOI:** 10.1080/23802359.2020.1765210

**Published:** 2020-05-14

**Authors:** Shaili Johri, Nicholas Dunn, Taylor K. Chapple, David Curnick, Vincent Savolainen, Elizabeth A. Dinsdale, Barbara A. Block

**Affiliations:** a Hopkins Marine Station, Stanford University, Pacific Grove, CA, USA; b Department of Biology, San Diego State University, San Diego, CA, USA; c Zoological Society of London, Institute of Zoology, London, UK; d Department of Life Sciences, Imperial College London, Ascot, UK; e Coastal Oregon Marine Experiment Station, Oregon State University, Newport, OR, USA

**Keywords:** Chagos, MPA, sharks, ecosystems, conservation, MinION, molecular taxonomy

## Abstract

The Chagos archipelago in the British Indian Ocean Territory (BIOT) has been lacking in detailed genetic studies of its chondrichthyan populations. Chondrichthyes in Chagos continue to be endangered through illegal fishing operations, necessitating species distribution and abundance studies to facilitate urgent monitoring and conservation of the species. Here, we present a complete mitochondrial genome of the Silvertip Shark, *Carcharhinus albimarginatus* sampled in the Chagos archipelago. The mitochondrial genome of *C. albimarginatus* was 16,706 bp in length and consisted of 13 protein-coding genes, 22 tRNA genes, two rRNA genes, a replication origin and a D-loop region. GC content was at 38.7% and the control region was 1,065 bp in length. We expect that mitogenomes presented here will aid development of molecular assays for species distribution studies. Overall these studies will promote effective conservation of marine ecosystemes in the BIOT.

The Silvertip shark (*Carcharhinus albimarginatus*) is a large requiem shark species with fragmented distribution in the tropical Indian and Pacific Oceans, and is currently listed as ‘Vulnerable’ on the IUCN Red List (González-Medina and Pillans 2015). It is found throughout the British Indian Ocean Territory Marine Protected Area (BIOT MPA), but is frequently caught by illegal fisheries (Ferretti et al. 2018; Tickler et al. 2019), leading to severe population declines locally (Ferretti et al. 2018).

We sequenced the mitochondrial genome of *C. albimarginatus*. Samples were obtained from two individuals in the BIOT in March 2018 (Specimen 1: Latitude: 07°08′50.1600″S; Longitude: 072°08′19.5000″E and Specimen 2: Latitude: 07°08′17.6400″S; Longitude: 072°11′48.1800″E). These specimens were stored at the Hopkins Marine Station, Stanford University (Sample Accession #0200022322322) and Silwood Park, Imperial College London (Sample Accession #020002086005) in 70% ethanol. DNA for Specimen 1 was extracted from the fin clip of a juvenile male and sequenced on the MinION handheld sequencer following (Johri et al. 2019). Ninety-five Fast5 files obtained from 72 h of sequencing were converted to FASTQ files using the Guppy 3.3.1 basecaller (Oxford Nanopore Technologies, ONT) and a GPU interface for 12 h post sequencing. A total of 374, 396 sequence reads were obtained from the sequencing run, with a length range of 350 to 65,000 base pairs (bp). The reads were trimmed and mapped as described previously (Johri et al. 2019). The resulting contig consisted of 115 mapped reads which were checked for nucleotide assignment and alignment errors, and highest Q scores were used to build consensus at ambiguous sites. The consensus sequence was annotated following (Johri et al. 2019; Johri et al. 2020). Second, genomic DNA was extracted from another juvenile male (specimen 2) using the Qiagen Blood & Tissue Kit and sequenced using an Illumina HiSeq platform. The mitochondrial genome sequence was then extracted bioinformatically yielding an almost identical sequence, which only differed from the sequence for Specimen 1 by 3 bp. These sequence differences include 3 single nucleotide polymorphisms which may be actual sequence differences or artifacts resulting from sequencing methods.

The mitochondrial genome of *C. albimarginatus* (GenBank: MT104516, MT093206) was 16,706 bp in length and consisted of 13 protein-coding genes (PCGs), 22 tRNA genes, two rRNA genes, a replication origin and a D-loop region. GC content was at 38.7%. All PCGs started with ATG and some PCGs ended with an incomplete stop codon. The control region was 1,065 bp in length.

To assess the phylogenetic position of *C. albimarginatus*, gene trees were constructed using mitogenomes from six families within the order Carcharhiniformes and Hexanchiformes was used as outgroup. Phylogenies were determined in Bayesian inference frameworks (Huelsenbeck and Ronquist 2001; Edgar 2004; Ronquist et al. 2012) following (Johri et al. 2019; Johri et al. 2020). Bayesian trees were estimated using the GTR substitution model, gamma rate variations with 4 gamma categories, chain length 110,000, burn-in length 100,000 and subsampling frequency 200. *C. albimarginatus* was nested within Carcharhinidae and most closely related to the *C. albimarginatus* reference (Figure 1).

**Figure 1.**
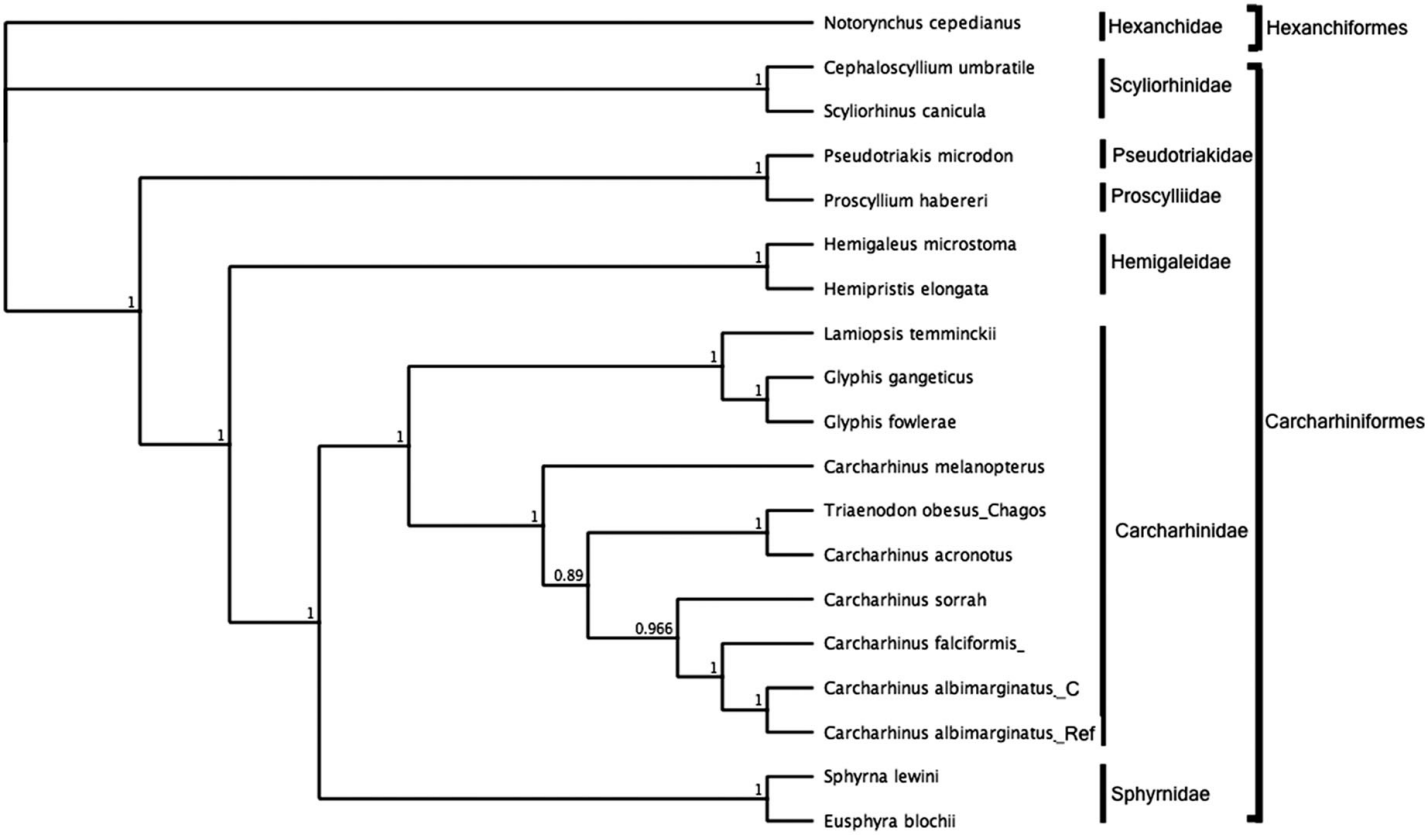
Bayesian estimate of phylogenetic position of *Carcharhinus albimarginatus* within the order Carcharhiniformes computed with complete mitochondrial genomes. Members of the order Hexanchiformes served as the outgroup. Families are indicated by vertical lines and orders by square brackets. Numbers at nodes are posterior probabilities. GenBank Accession Numbers: *Notorynchus cepedianus* (AB560489.1); *Cephaloscyllium umbratile* (KT003686.1); *Scyliorhinuscanicula* (Y16067.1); *Proscylliumhabereri* (KU721838.1); *Pseudotriakismicrodon* (AB560493.1); *Hemipristiselongata* (KU508621.1); *Hemigaleusmicrostoma* (KT003687.1); *Lamiopsistemminckii* (KT698048.1); *Glyphisfowlerae* (KT698049.1); *G. gangeticus* (KT698040.1); *Carcharhinusmelanopterus* (KJ720818.1); *C. sorrah* (KF612341.1); *C.falciformis*(MK092088); *C. acronotus* (KF728380.1); *Carcharhinus albimarginatus* _Ref (JQ518609.1); *C. albimarginatus*_C (MT104516); *Eusphyrablochii* (KU892590.1); *Sphyrna lewini* (JX827259.1).

Mitogenomes presented here will enable identification of interspecies genetic divergence through PCR amplification and accurate species identification using environmental DNA sampling. The current research findings will enable substantial conservation research and management of Chondrichthyes in the BIOT MPA and in other marine ecosystems.

## Data Availability

Data that support the findings of this study are openly available in Genbank with reference accession numbers MT104516 and MT093206 at DOI: https://www.ncbi.nlm.nih.gov/nuccore/MT104516.1 and https://www.ncbi.nlm.nih.gov/nuccore/MT093206.1, respectively.
